# Prevalence and antimicrobial resistance of coagulase negative staphylococci clinical isolates from Ethiopia: a meta-analysis

**DOI:** 10.1186/s12866-018-1188-6

**Published:** 2018-05-25

**Authors:** Serawit Deyno, Sintayehu Fekadu, Sisay Seyfe

**Affiliations:** 1 PHARMBIOTRAC, Mbarara University of Science and Technology, Mbarara, Uganda; 2 Department of Pharmacology, School of Medicine, College of Medicine and Health Sciences, Hawassa University, P. O. Box 1560, Hawassa, Ethiopia; 3 Department of Microbiology, Faculty of Medicine, Shimane University, Shimane, Japan; 4 Department of Biochemistry, School of Medicine, College of Medicine and Health Sciences, Hawassa University, Hawassa, Ethiopia

**Keywords:** Antimicrobial resistance, Methicilin, Vancomycin, CoNS, Meta-analysis, Ethiopia

## Abstract

**Background:**

Antimicrobial resistant Coagulase-negative Staphylococci (CoNS) have limited treatment options, rendered diseases untreatable and made hospitals to be reservoirs of the resistant strains. The aim of this study was to estimate the pooled prevalence and antimicrobial resistance of clinical isolates of CoNS from Ethiopia.

**Results:**

The electronic database search yielded 6511 articles of which 21 met predefined inclusion criteria. The pooled prevalence of CoNS from Ethiopia was 12% (95% confidence interval (CI): 8, 16%). The analyses revealed high level of CoNS resistance to methicilin (37%[95% CI: 21, 55%]), vancomycin (911%[95% CI: 0, 35%]), penicillin (58%[95% CI: 42, 74%]), amoxicillin (42%[95% CI: 23, 61%]), amoxicillin-clavulanate (27%[95% CI: 2, 27%]), ampicillin (64%[95% CI: 46, 80%]), tetracycline (60% [95% CI: 49, 70%]), doxycycline (36%[95% CI:19,55%]), Sulfamethoxazole-trimethoprim (50%[95% CI: 36, 64%]), ceftriaxone (27% [95% CI: 18, 38%]), cephalothin (32% [95% CI: 7, 62%]), norfloxacin (39%[95% CI: 24, 56%]), chloramphenicol (40%[95% CI: 23, 58%]), clindamycin (11% [95% CI: 2, 27%]), ciprofloxacin (14%[95% CI: 6, 22%]), gentamicin (27%[95% CI:19,36%]) and erythromycin (30%[95% CI:20%,42%]). High heterogeneity, I^2^ ranging from 69.04 to 96.88%; *p*-values ≤0.01, was observed. Eggers’ test did not detect publication bias for the meta-analyses and low risk of bias was observed in included studies.

**Conclusions:**

CoNS has gotten resistant to commonly used antimicrobials from Ethiopia. There is a need of launching national antimicrobial treatment, development and implementation of policy guidelines to contain the threat. Further research focusing on factors promoting resistance and the effect of resistance on treatment outcome studies are warranted.

**Electronic supplementary material:**

The online version of this article (10.1186/s12866-018-1188-6) contains supplementary material, which is available to authorized users.

## Background

The Coagulase-negative Staphylococci (CoNS) are normal flora which often cause infection associated with implanted appliances and devices, especially in the old, very young children and immune-compromised patients [1, 2]. On human body, there is a widespread distribution of CoNS from normal flora to those that cause severe diseases [3, 4]. Acquisition of genes result in the conversion of commensal staphylococci into invasive pathogens [5]. The vast majority of infections caused by CoNS results in hospitalization [6] and they are among the five most commonly reported pathogens in nosocomial infections [7]. Many of the CoNS species are commonly resistant to antimicrobials used currently against staphylococcal infections [8, 9]. The spread of multi-drug resistant CoNS strains has been promoted by the use of antibiotics in hospitals which has provided a reservoir of antimicrobial resistant strains. The genetic exchange between CoNS and *Staphylococcus aureus (S. aureus)* and the widespread prevalence of methicillin resistance among CoNS species is a great public health concern [5, 9, 10].

The rising trend of antimicrobial resistant staphylococci species including CoNS [11–15] worldwide requires national antimicrobial resistance prevention policy and updated treatment guidelines which are based on national antimicrobial resistance surveillance. However, developing countries like Ethiopia have financial constraints to conduct national antimicrobial resistance survey. Small scale studies funded by various organizations are conducted and these studies could provide ample evidence to fulfill the gap in national antimicrobial surveillance data if they are summarized and synthesized to draw national estimate of national antimicrobial resistance prevalence. To this end, we had previously conducted a meta-analysis of resistance of *S. aureus* to antimicrobial agents in Ethiopia [16]. The finding of the study revealed a very high level of *S. aureus* resistance to almost to all of antimicrobial agents commonly used in Ethiopia. Similarly, CoNS are important opportunistic pathogens showing a growing threat of antimicrobial resistance and becoming a difficult-to- treat pathogens. There is a need to have summarized evidence on AMR burden among CoNS to draw national estimate of CoNS antimicrobial resistance prevalence. The current study focuses on CoNS species while the previous study entirely focuses on *S. aureus* [16]*.* The two studies are separate and there is no overlapping data. However the two studies followed similar study design and objectives.

To the best of our knowledge, there is neither nationwide prevalence surveillance and resistance data nor meta-analysis or systematic review on prevalence of antimicrobial resistance of CoNS in Ethiopia. However, many small scale studies are conducted in many parts of the country. The studies had clear differences in setting, population, methodology, findings, and other characteristics. The purpose of this study was, therefore, to determine prevalence and antimicrobial resistance of CoNS using the best available literature from Ethiopia.

## Methods

### Study design

Meta-analysis of prevalence and antimicrobial resistance of CoNS was conducted using the best available evidence from Ethiopia.

### Literature search strategy

To identify potentially eligible studies, databases of Pub Med, Google Scholar, Hinari, Scopus and directory of open Access Journals (DOAJ) were searched until December 2016. Two of the authors (SS and SF) independently searched for relevant studies to be included. Selection of the study was done by the two authors independently. Consensus was reached on discussion with the third author (SD) whenever disagreement arose. Endnote software was used to manage the references. Articles indexed in Pub Med were directly downloaded using Endnote while those not found in Pub Med are manually added to Endnote. The reference lists of the identified studies were manually searched to identify additional relevant studies for inclusion.

The search was done using various key words combined by Boolean search conjunctions ‘AND’, ‘OR’ and ‘NOT’. These combined key words are, ‘Staphylococci’ AND ‘antimicrobial resistance AND Ethiopia’, ‘Staphylococci’ AND ‘antimicrobial susceptibility’ AND ‘Ethiopia’, ‘Staphylococci’ AND ‘antibiotic resistance’ AND ‘Ethiopia’, ‘Staphylococci’ AND ‘antibiotic susceptibility’ AND ‘Ethiopia’, ‘Staphylococci’ AND ‘drug resistance’ AND ‘Ethiopia’, ‘Staphylococci’ AND ‘drug susceptibility’ AND ‘Ethiopia’, “Staphylococci’ AND ‘antibacterial resistance’ AND ‘Ethiopia’, ‘Staphylococci’ And ‘antibacterial susceptibility’ AND ‘Ethiopia’.

#### Study selection procedures and criteria

Two-stage selection of the articles was conducted independently by two of the authors (SS and SF). In the first stage, the titles and abstracts of all retrieved articles were reviewed and grouped as eligible when they address the study question, otherwise dropped from further review. In the second stage, eligible articles were reviewed in full detail for decision on inclusion.

#### Eligibility criteria

Articles were selected based on predefined inclusion criteria. Included articles in this study were those that had the following characteristics: Prospective or retrospective studies, original journal articles, with antimicrobial susceptibility test data according to the criteria of the Clinical Laboratory Standards Institute (CLSI) [17], studies which defined antimicrobial resistance range according to CLSI manual, and those that used only clinical isolates. Duplicate studies, studies with small number of isolates (1–9), studies conducted other than clinical subjects like on foods, food handlers’ belongings, health workers’ belongings, health workers’ carriage or animals and of non-infectious carriage were excluded.

#### Data extraction

Excel spreadsheets prepared by SD were used for data extraction. The characteristics of studies extracted included first author name, year of publication, place of study, study design, total number of CoNS, number of resistant CoNS isolates, and isolation source. The primary outcome of this study was prevalence and antimicrobial resistant CoNS. If the proportion of sensitive isolates (x) was reported, the number of resistant isolates was calculated by multiplying the number of total isolates (y) by one minus the proportion of sensitive isolates (1-x). The proportion of CoNS is obtained from the division of CoNS positive population by total population under the study.

#### Risk of bias assessment

The methodological quality of each included study was assessed using the quality assessment checklist for prevalence studies as used in the study [18]. Graphs of the summary of the risk of bias were developed using RevMan 5.3 (Cochrane Informatics and Knowledge Management Department, London, UK).

#### Statistical analysis

Statistical analyses were conducted using Stata version 13.0 (Statacorp, LP, college station, TX). The prevalence values were pooled using the *metaprop* command in Stata [19]. Heterogeneity of the studies was assessed using the I^2^ statistic. Because of significant heterogeneity amongst the studies, the random-effects model (REM) was used to estimate the pooled proportion and 95% CIs using the DerSimonian and Laird method [20]. The Freeman-Turkey double arcsine transformation was used to avoid the missing of proportions near or at 0 and 1 from meta-analysis [21]. Subgroup analysis was done by study design, isolation source and study area. The presence of publication bias was tested using Egger’s test [22]. This meta-analysis was conducted as per the Preferred Reporting Items for Systematic Reviews and Meta-Analysis (PRISMA) guidelines [23].

## Results

### Included studies and characteristics

**Fig. 1:**
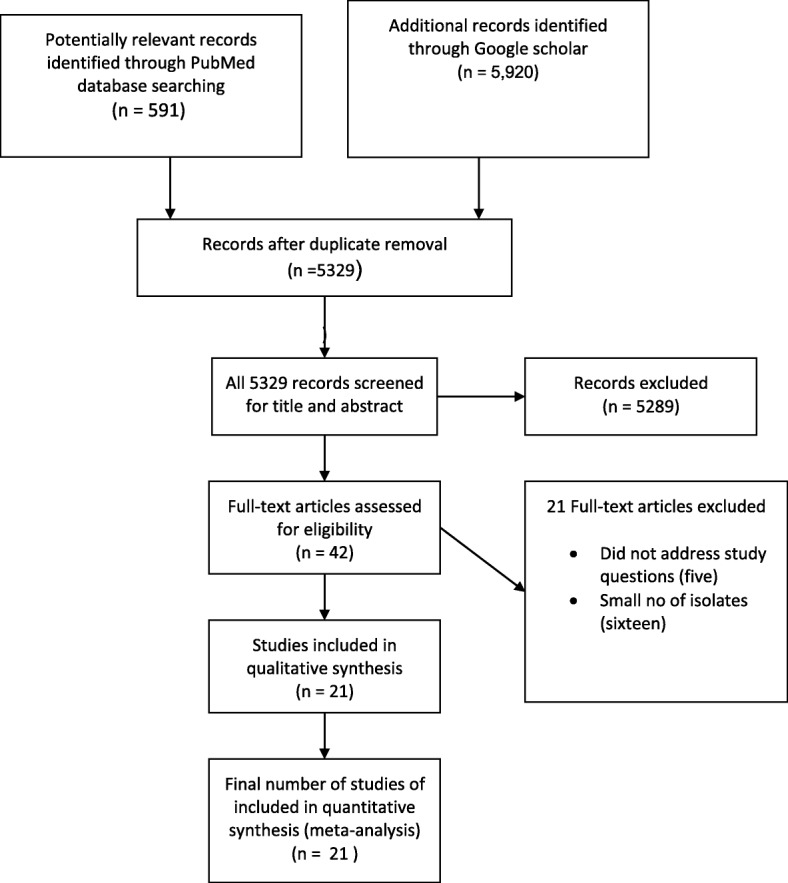
Flow diagram of retrieval of studies: Number of studies screened, assessed for eligibility, and included in the meta-analysis with reasons

Pub Med database search yielded 591, and Google database yielded 5920. After removal of the duplications, 5329 articles remained for further examination. Title and abstract screening reduced the number of eligible articles to 42 for full text evaluation. Twenty-one articles [13–15, 24–41] satisfied the criteria for inclusion for meta-analysis and twenty-one studies were excluded with reasons. Five studies did not address the study question (proportion of resistant CoNS) and were excluded [42–46]. The other sixteen studies with small number of isolates (less than 10) [47–59] were also excluded (Fig. 1).

Among total of 8047 patients who tested for CoNS, 647 were positives and the isolates were from ear discharge, eye discharge, blood, wound infection, surgical site infection, mixed samples and urine sample. Eleven studies were primary data [13, 15, 25, 27, 28, 30, 36–40] while nine studies were secondary data (records from hospitals or regional laboratories) [14, 24, 26, 29, 31–35]. The characteristics of included studies were summarized in Table 1. Identification of CoNS was conducted using culture on blood agar, Gram-staining, catalase/coagulase test, and cocktail of biochemical assays for carbohydrate fermentation or utilization of specific substrate in all included studies. Kirby Bauer disk diffusion method was used for determination of antimicrobial susceptibility test.

**Table 1:** Characteristics of the studies included in the meta-analyses

No	Study	Study period	Study area	Isolate source	Data type	Sample size	No of CoNS positive samples
1.	Hailu et al. 2016 [24]	2013–2015	Bahirdar	Ear discharge	Secondary	368	34
2.	Abera et al. 2008 [25]	2006	Bahirdar	SSI, wound, ear and eye discharges and throat swabs	Primary	221	59
3.	Biadglegn et al. 2009 [26]	2003–2008	Bahirdar	Urine	Secondary	529	10
4.	Mama et al. 2014 [15]	2013	Jimma	Wound	Primary	145	21
5.	Shiferaw et al. 2015 [13]	2014	Dessie	Eye discharges	Primary	160	51
6.	Sewunet et al. 2013 [36]	2010	Adiss Ababa	Wound	Primary	50	15
7.	Guta et al. 2014 [27]	2010–2011	Hawassa	SSI	Primary	100	26
8.	Mengesha et al. 2014 [28]	2012	Mekele	SSI	Primary	128	18
9.	Kibret and Abera 2010 [34]	2003–2010	Dessie	urine, ear discharge, eye discharge and wound swab	Secondary	3149	33
10.	Kibret and Abera 2014 [35]	2003–2010	Dessie	Urine	Secondary	1404	17
11.	Tenssay 2002 [38]	1997–1998	Jimma	Pus, blood, urine, and stool samples	Primary	545	89
12.	Wasihun et al. 2015 [41]	2014–2015	Mekele	Ear discharge	Secondary	514	44
13.	Lema et al. 2012 [39]	2006–2007	Addis Ababa	Wound	Primary	245	68
14.	Dagnew et al. 2013 [29]	2006–2012	Gondar	Blood	Secondary	390	30
15.	Godebo et al. 2013 [30]	2011	Jimma	Wound	Primary	322	14
16.	Muluye et al. 2013 [31]	2009–2012	Gondar	Ear discharge	Secondary	228	23
17.	Muluye et al. 2014 [14]	2009–2012	Gondar	Eye discharges	Secondary	102	17
18.	Tesfaye et al. 2013 [40]	2012-O2012	Jimma	Eye discharges	Primary	198	15
19.	Tadesse et al. 2014 [37]	2012	Hawassa	Urine	Primary	244	19
20.	Aweke et al. 2014 [33]	2012–2013	Hawassa	Eye discharges	Secondary	281	26
21.	Washun and Zemen 2015 [41]	2014–2015	Mekele	Ear discharges	Primary	162	17

### Publication bias, heterogeneity and risk of bias assessment

**Fig. 2:**
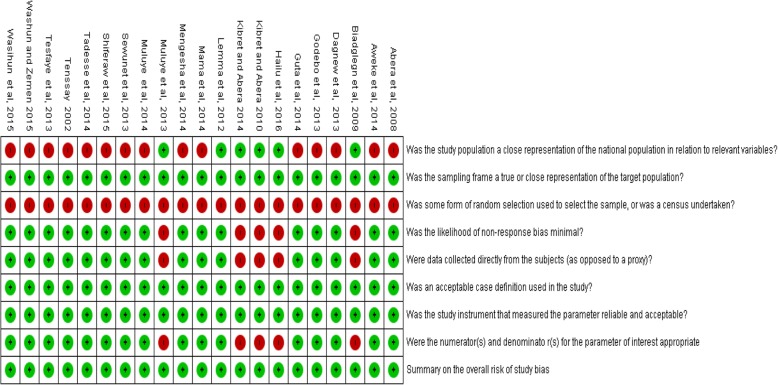
Summary of risk of bias for all the included studies

High heterogeneity, I^2^ ranging from 67.65 to 96.76%; *p*-values ≤0.01 was observed. Eggers’ test didn’t detect publication bias. Overall, most of the prospective studies demonstrated a low risk of bias; however that of retrospective relatively a higher risk of bias. The methodological quality of the studies included is illustrated in (Fig. 2).

### Prevalence of coagulase-negative staphylococci

**Fig. 3:**
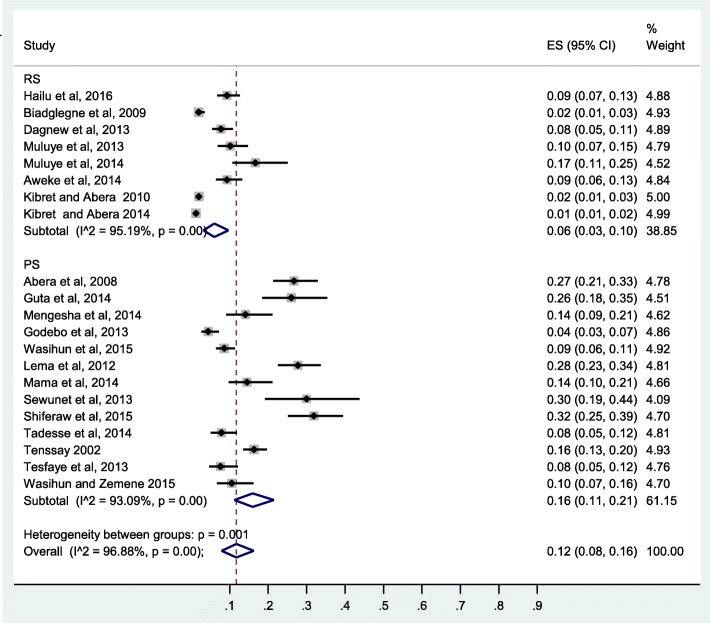
Forest plot of prevalence of CoNS from Ethiopia by study design

**Fig. 4:**
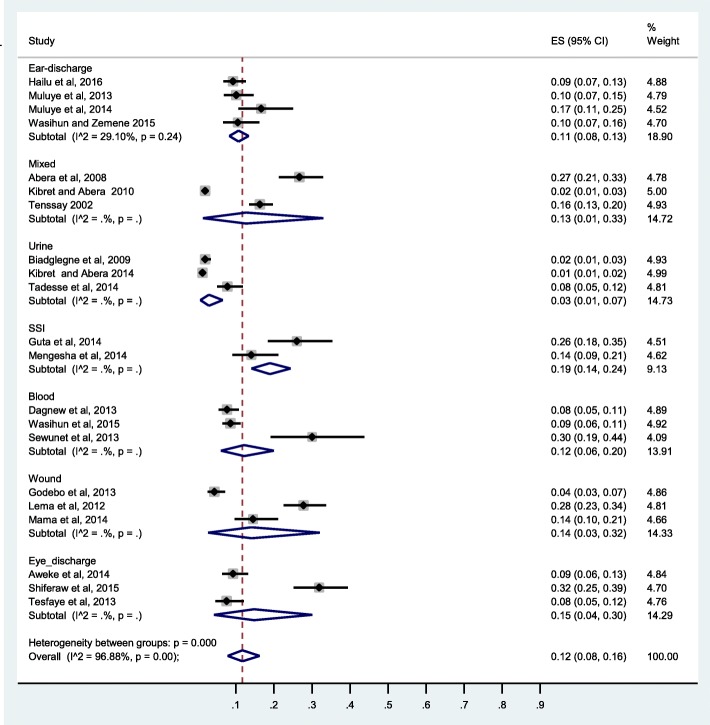
Forest plot of prevalence of CoNS from Ethiopia by isolation source

**Fig. 5:**
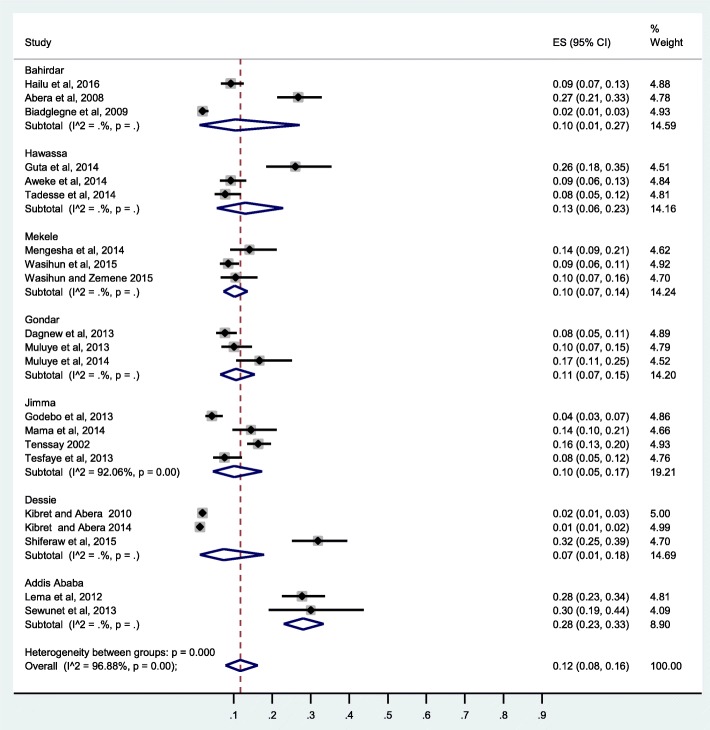
Forest plot of prevalence of CoNS from Ethiopia by area (region)

The pooled prevalence of CoNS clinical isolates from Ethiopia was 12%(95% CI: 8, 16%), I2 = 96.88%. Subgroup analysis by study design showed significantly higher prevalence of CoNS in prospective studies compared to retrospective studies (16%(95% CI: 11, 21%) versus 6%(95% CI: 3, 10%)) (Fig. 3). Higher prevalence from surgical site infection (SSI) (19%) was observed compared to urinary tract infection (3%) (Fig. 4). A subgroup analysis by study region showed a higher value of CoNS in Addis Ababa 28%, significantly higher than Mekelle, Jimma, Dessie, and Gondar (Fig. 5).

### Prevalence of antimicrobials resistant coagulase-negative staphylococci

High level of resistance by CoNS was observed to most commonly used antimicrobials from Ethiopia and the resistance level was summarized in Table 2. Methicilin resistance was 37% (95 %CI: 21, 55%), Additional file 1: Figure S1; to vancomycin 11% (95% CI: 0, 35%), Additional file 2: Figure S2; to penicillin 58%(95% CI: 42, 74%), Additional file 3: Figure S3; to amoxicillin 42% (95% CI: 23, 61%), Additional file 4: Figure S4; to amoxicillin-clavulanate 27%(95% CI: 2, 27%), Additional file 5: Figure S5; to ampicillin 64%(95% CI: 46, 80%), Additional file 6: Figure S6. Similarly, resistance to other antimicrobials was high; to tetracycline 60% (95% CI: 49, 70%), Additional file 7: Figure S7; to doxycycline 36% (95% CI:19,55, 81.18%, Additional file 8: Figure S8; to sulfametaxazole-trimethoprim 50% [95% CI: 36, 64%), Additional file 9: Figure S9; to ceftriaxone (27% [95% CI: 18, 38%]), Additional file 10: Figure S10; to cephalothin (32% [95% CI: 7, 62%], Additional file 11: Figure S11; to norfloxacin (39% [95% CI: 24, 56%], Additional file 12: Figure S12; to chloramphenicol (40% [95% CI: 23, 58%], Additional file 13: Figure S13; to clindamycin (11% [95% CI: 2, 27%]) Additional file 14: Figure S14; and to ciprofloxacin (14% [95% CI: 6, 22%]), Additional file 15: Figure S15; to gentamicin 27% (95%, CI:19, 36%), Additional file 16: Figure S16; and to erythromycin 30% (95%, CI:20, 42%) Additional file 17: Figure S17. Forest plots of the abovementioned antimicrobials resistance were placed as additional file respectively from methicilin to erythromycin (Additional file 1: Figure S1, Additional file 2: Figure S2, Additional file 3: Figure S3, Additional file 4: Figure S4, Additional file 5: Figure S5, Additional file 6: Figure S6, Additional file 7: Figure S7, Additional file 8: Figure S8, Additional file 9: Figure S9, Additional file 10: Figure S10, Additional file 11: Figure S11, Additional file 12: Figure S12, Additional file 13: Figure S13, Additional file 14: Figure S14, Additional file 15: Figure S15, Additional file 16: Figure S16 and Additional file 17: Figure S17) and the proportions were summarized in Table 2.

**Table 2:** Prevalence of CoNS resistance to different antimicrobial agents in Ethiopia

	Antimicrobial Agent	Frost plot presented in	No. of studies	No. of isolate tested	No. of resistant isolate	Pooled AMR Proportion (95% CI)	I^2^ (*p*-value)
1.	Methicilin	S1	9	317	140	0.37 (0.21,0.55)	88.70 (*P* ≤ 0.01)
2.	Vancomycin	S2	7	169	24	0.11 (0.00,0.35)	92.54 (*P* ≤ 0.01)
3.	Penicillin	S3	14	389	225	0.57 (0.40,0.73)	89.60 (*P* ≤ 0.01)
4.	Amoxicillin	S4	9	198	81	0.42 (0.23,0.61)	84.37 (P ≤ 0.01)
5.	Amoxicillin-clavulanate	S5	5	120	33	0.27 (0.02,0.65)	93.77 (*P* ≤ 0.01)
6.	Ampicillin	S6	13	338	216	0.64 (0.46,0.80)	89.92 (*P* ≤ 0.01)
7.	Tetracycline	S7	15	388	239	0.60 (0.49,0.70)	76.30(*P* ≤ 0.01)
8.	Doxycycline	S8	6	162	63	0.36 (0.19,0.55)	81.18(*P* ≤ 0.01)
9.	Sulfametaxazole-trimethoprim	S9	16	413	207	0.50 (0.36,0.64)	86.95(*P* ≤ 0.01)
10.	Ceftriaxone	S10	14	317	95	0.27 (0.18,0.38)	71.91 (*P* ≤ 0.01)
11.	Cephalothin	S11	5	61	23	0.32 (0.07,0.62)	78.01 (*P* ≤ 0.01)
12.	Norfloxacin	S12	8	177	58	0.39 (0.24,0.56)	74.57 (*P* ≤ 0.01)
13.	Chloramphenicol	S13	12	311	141	0.40 (0.23,0.58)	88.94(*P* ≤ 0.01)
14.	Clindamycin	S14	5	209	39	0.11 (0.02,0.27)	85.72 (*P* ≤ 0.01)
15.	Ciprofloxacin	S15	13	316	51	0.14 (0.06,0.22)	73.49 (*P* ≤ 0.01)
16.	Gentamicin	S16	17	431	123	0.27 (0.19,0.36)	69.04 (*P* ≤ 0.01)
17.	Erythromycin	S17	16	413	138	0.30 (0.20,0.42)	81.12 (*P* ≤ 0.01)

**Fig. 6:**
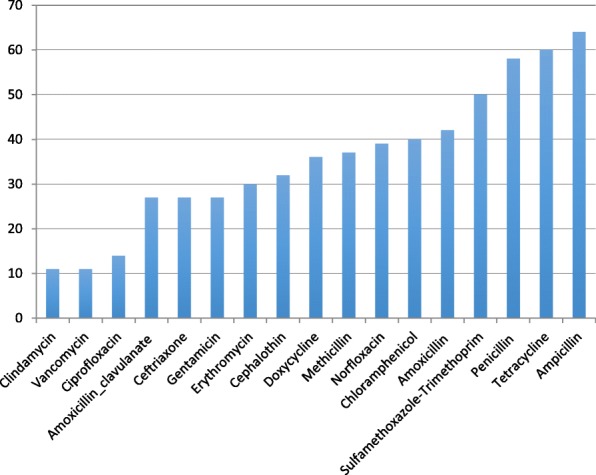
Comparison of prevalence of CoNS resistance to different antimicrobials from Ethiopia

Comparisons of the prevalence of CoNS resistance to different antimicrobial agents were outlined in (Fig. 6). The magnitude of CoNS resistance to the different antimicrobials ranges from 11% to vancomycin and clindamycin to 64% to ampicillin.

## Discussion

In this meta-analysis, we estimated the pooled prevalence and antimicrobial resistance of CoNS to 17 different antimicrobials commonly used in Ethiopia. Twenty-one studies were included in this meta-analysis and the number of studies included in each meta-analysis varies from 5 to 17. Overall, the 21 studies provided evidence concerning the prevalence and antimicrobial resistance of CoNS to different antimicrobials based on isolates identified from 8047 patients. The study revealed that CoNS resistances to commonly available antimicrobials were high, ranging from 11% (vancomycin) to 64% (clindamycin and amipicilin) in Ethiopia.

The emergence of antimicrobial resistant staphylococcus species is a global concern. Our previous studies showed that MRSA is unexpectedly high (97.0%) in Hawassa University comprehensive specialized hospital, Ethiopia [12] and meta-analysis on MRSA in Ethiopia demonstrated very high level of resistance (47% [95% CI: 33, 61%]) [16] and in other meta-analysis similar results were observed (32.5% [95% CI, 24.1 to 40.9%]) [60]. The current findings on MRCoNS showed similarly high resistance (34 95% CI [17, 53%]). These findings signified that methicillin resistance is increasing and becoming of public health concern in Ethiopia. The prevalence of MRCoNS was relatively lower than MRSA. This may be due to infrequent occurrence of infection with CoNS compared to *S. aureus* resulting in decreased antimicrobial exposure. However, CoNS is continuously evolving from commensal staphylococci into invasive pathogens and then to resistant strains and possibly gaining resistant genes from *S. aureus* [5].

There is no published meta-analysis focusing on antimicrobial resistance of CoNS for comparison, however the pooled prevalence of CoNS resistance in this study showed a higher level of resistance compared to other studies in different parts of the world [8, 61]. A higher prevalence of resistance was observed in a study conducted in India compared to the current study [62, 63]. A higher rate of resistance in this study may be due to a higher exposure, irrational use of antimicrobial agents and lack of infection prevention policies, especially in hospital settings.

Subgroup analysis by study design showed a higher pooled prevalence of CoNS in prospective studies (17%) compared to retrospective studies (6%). This could be attributed to missing data and poorly defined denominator in the case of retrospective design. A higher prevalence of CoNS in SSI may be due to the fact that CoNS are opportunistic pathogens causing infections in patients with implanted medical devices and surgical procedures [8, 64]. A higher prevalence of CoNS in Addis Ababa compared to other regions can be explained by lager exposure rate in Tikur Anbessa referral hospital where referral cases with severe diseases of recurrent infection treated from all over the country.

Aminoglycosides and fluoroquinolones showed relatively lower level of resistance in this study. A lower level of resistance observed could suggest the development of mutant resistant strains for commonly used first line agents made the microbes easily susceptible to less commonly used antimicrobials [65, 66]. The lower rate of resistance observed with clindamycin may be due to its infrequent use in Ethiopia resulting in lower exposure rate.

The cause of antimicrobial resistance is multifactorial, from lack of infection prevention to irrational use of antimicrobials by health professionals and patients [67]. It is a common practice that antimicrobials can be obtained over-the-counter in Ethiopia. This mishandling of antimicrobials is the main cause of emergence of resistance [68]. Absence of culture techniques and routine antimicrobial susceptibility testing and consequent empiric therapy is a reason for selection of resistant strains and spread. Therefore, in line to strategies for prevention and containment of resistance there is a need for innovative ways of halting resistance. Combination therapy and search for novel antimicrobials will provide a vital role to counter this global problem.

Significant risk of bias was observed with five retrospective studies. This risk of bias was due to unclear denominator in retrospective study during data retrieval and possibly loss of samples during retrieval resulting in non-response. In addition, all of the included studies were conducted in small particular localities and non-representative convenient samplings technique was used. Therefore, the limitations of this study arise from the characteristics of included studies. First, this is in vitro antimicrobial resistance testing and direct inference to clinical outcome calls for caution. Secondly, many studies were conducted in limited localities and mainly in teaching hospitals in bigger cities where patients with advanced and severe stages of diseases with recurrent infection are treated. For this reason, the resistance prevalence could have been overrated. Lastly, studies were not conducted on phenotypic characteristics of resistance, lacking details of molecular characteristic of resistant strains.

## Conclusions

This meta-analysis revealed that CoNS has gotten resistant to many of common antibiotics used in Ethiopia. Launching national antimicrobial treatment and use policy guideline is essential for fighting the antimicrobial resistance. Further research focusing on factors promoting antimicrobial resistance, molecular genetics and outcome studies are warranted. Antimicrobial susceptibility should be determined prior to treatment of infections.

## Additional files

Additional file 1: **Figure S1.** Forest plot of the proportion of CoNS resistance to methicillin. (DOCX 19 kb)

Additional file 2: **Figure S2.** Forest plot of the proportion of CoNS resistance to vancomycin. (DOCX 16 kb)

Additional file 3: **Figure S3.** Forest plot of the proportion of CoNS resistance to penicillin. (DOCX 19 kb)

Additional file 4: **Figure S4.** Forest plot of the proportion of CoNS resistance to amoxicillin. (DOCX 18 kb)

Additional file 5: **Figure S5.** Forest plot of the proportion of CoNS resistance to amoxicillin-clavulanate. (DOCX 17 kb)

Additional file 6: **Figure S6.** Forest plot of the proportion of CoNS resistance to amipicillin. (DOCX 18 kb)

Additional file 7: **Figure S7.** Forest plot of the proportion of CoNS resistance to tetracycline. (DOCX 20 kb)

Additional file 8: **Figure S8.** Forest plot of the proportion of CoNS resistance to doxycycline. (DOCX 16 kb)

Additional file 9: **Figure S9.** Forest plot of the proportion of CoNS resistance to sulfamethoxazole-trimethoprim. (DOCX 21 kb)

Additional file 10: **Figure S10.** Forest plot of the proportion of CoNS resistance to ceftriaxone. (DOCX 20 kb)

Additional file 11: **Figure S11.** Forest plot of the proportion of CoNS resistance to cephalothin. (DOCX 17 kb)

Additional file 12: **Figure S12.** Forest plot of the proportion of CoNS resistance to norfloxacin. (DOCX 18 kb)

Additional file 13: **Figure S13.** Forest plot of the proportion of CoNS resistance to chloramphenicol. (DOCX 18 kb)

Additional file 14: **Figure S14.** Forest plot of the proportion of CoNS resistance to clinidamycin. (DOCX 17 kb)

Additional file 15: **Figure S15.** Forest plot of the proportion of CoNS resistance to ciprofloxacin. (DOCX 19 kb)

Additional file 16: **Figure S16.** Forest plot of the proportion of CoNS resistance to gentamicin. (DOCX 21 kb)

Additional file 17: **Figure S17.** Forest plot of the proportion of CoNS resistance to erythromycin. (DOCX 21 kb)

## Abbreviations

CI: Confidence interval
CLSI: Clinical Laboratory Standards Institute
CoNS: Coagulase negative Staphylococci
MRCoNS: Methicillin-resistant coagulase-negative staphylococci
MRSA: Methicillin resistant *Staphylococcus aureus*
*S. aureus*: *Staphylococcus aureus*
SSI: Surgical sit infection
VRSA: Vancomycin resistant *Staphylococcus aureus*
