# Supplementary material for: Prevalence and antimicrobial resistance of coagulase negative staphylococci clinical isolates from Ethiopia: a meta-analysis
Source: BMC Microbiol. 2018 May 25;18:43. doi: 10.1186/s12866-018-1188-6 (PMC5970528; doi:10.1186/s12866-018-1188-6)
Supplement: Supplementary file 2 [file 12866_2018_1188_MOESM11_ESM.docx]

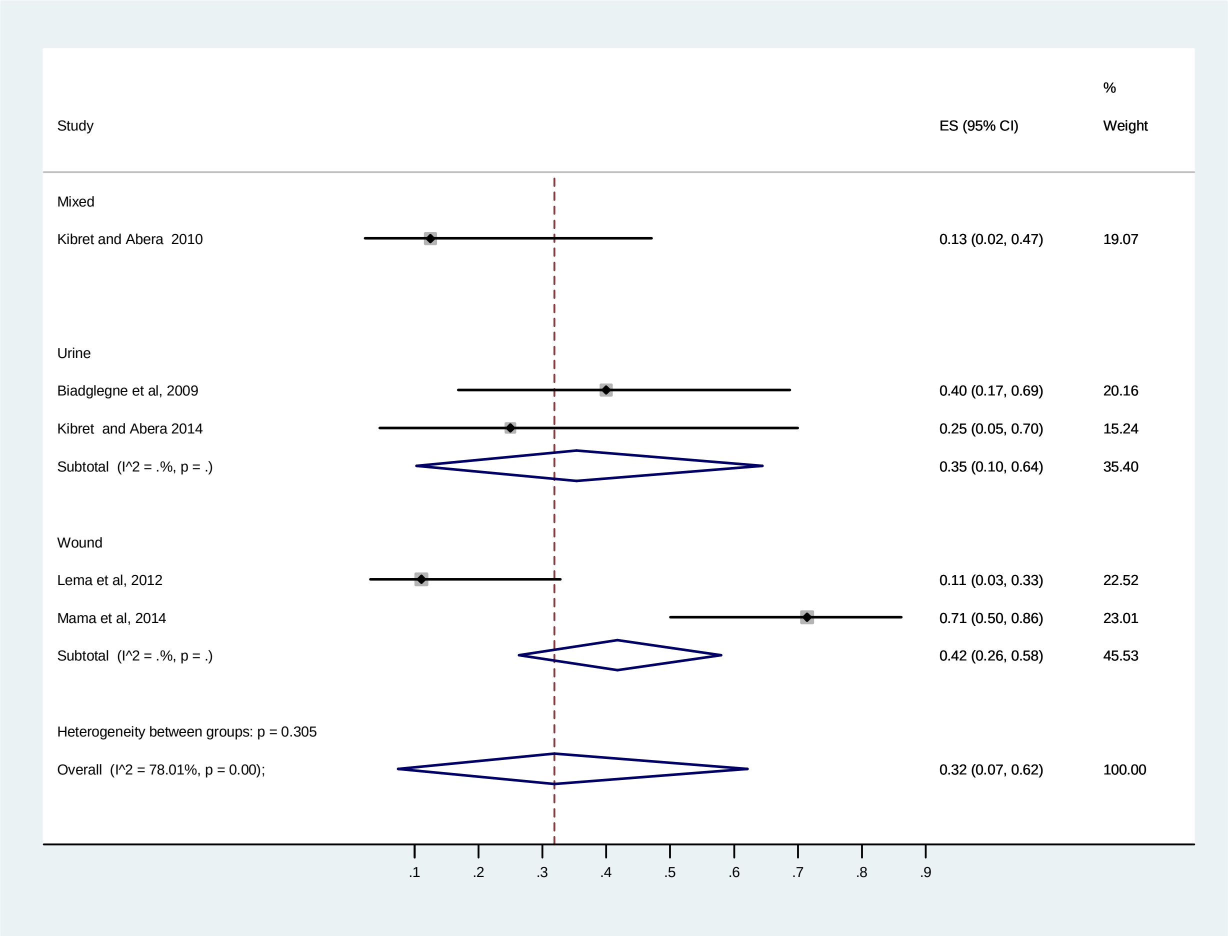
Study
ES (95% CI)
%
Weight
Mixed
Kibret and Abera 2010
0.13 (0.02, 0.47)
19.07
Urine
Biadglegne et al, 2009
0.40 (0.17, 0.69)
20.16
Kibret and Abera 2014
0.25 (0.05, 0.70)
15.24
Subtotal (I^2 = .%, p = .)
0.35 (0.10, 0.64)
35.40
Wound
Lema et al, 2012
0.11 (0.03, 0.33)
22.52
Mama et al, 2014
0.71 (0.50, 0.86)
23.01
Subtotal (I^2 = .%, p = .)
0.42 (0.26, 0.58)
45.53
Heterogeneity between groups: p = 0.305
Overall (I^2 = 78.01%, p = 0.00);
0.32 (0.07, 0.62)
100.00
.1
.2
.3
.4
.5
.6
.7
.8
.9
