# Supplementary material for: Prevalence and antimicrobial resistance of coagulase negative staphylococci clinical isolates from Ethiopia: a meta-analysis
Source: BMC Microbiol. 2018 May 25;18:43. doi: 10.1186/s12866-018-1188-6 (PMC5970528; doi:10.1186/s12866-018-1188-6)
Supplement: Supplementary file 3 [file 12866_2018_1188_MOESM12_ESM.docx]

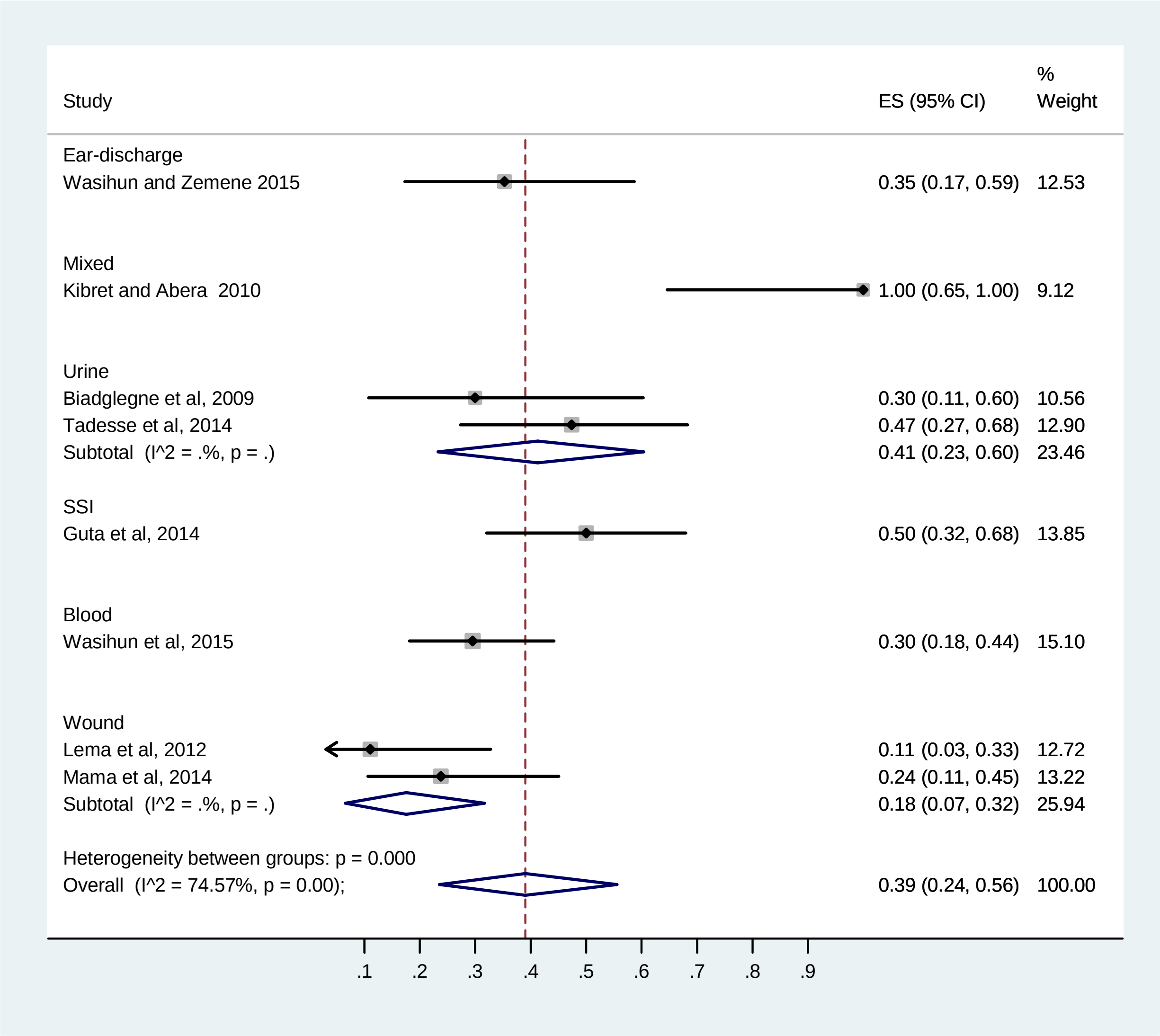
Study
ES (95% CI)
%
Weight
Ear-discharge
Wasihun and Zemene 2015
0.35 (0.17, 0.59)
12.53
Mixed
Kibret and Abera 2010
1.00 (0.65, 1.00)
9.12
Urine
Biadglegne et al, 2009
0.30 (0.11, 0.60)
10.56
Tadesse et al, 2014
0.47 (0.27, 0.68)
12.90
Subtotal (I^2 = .%, p = .)
0.41 (0.23, 0.60)
23.46
SSI
Guta et al, 2014
0.50 (0.32, 0.68)
13.85
Blood
Wasihun et al, 2015
0.30 (0.18, 0.44)
15.10
Wound
Lema et al, 2012
0.11 (0.03, 0.33)
12.72
Mama et al, 2014
0.24 (0.11, 0.45)
13.22
Subtotal (I^2 = .%, p = .)
0.18 (0.07, 0.32)
25.94
Heterogeneity between groups: p = 0.000
Overall (I^2 = 74.57%, p = 0.00);
0.39 (0.24, 0.56)
100.00
.1
.2
.3
.4
.5
.6
.7
.8
.9
