# Supplementary material for: Prevalence and antimicrobial resistance of coagulase negative staphylococci clinical isolates from Ethiopia: a meta-analysis
Source: BMC Microbiol. 2018 May 25;18:43. doi: 10.1186/s12866-018-1188-6 (PMC5970528; doi:10.1186/s12866-018-1188-6)
Supplement: Supplementary file 4 [file 12866_2018_1188_MOESM13_ESM.docx]

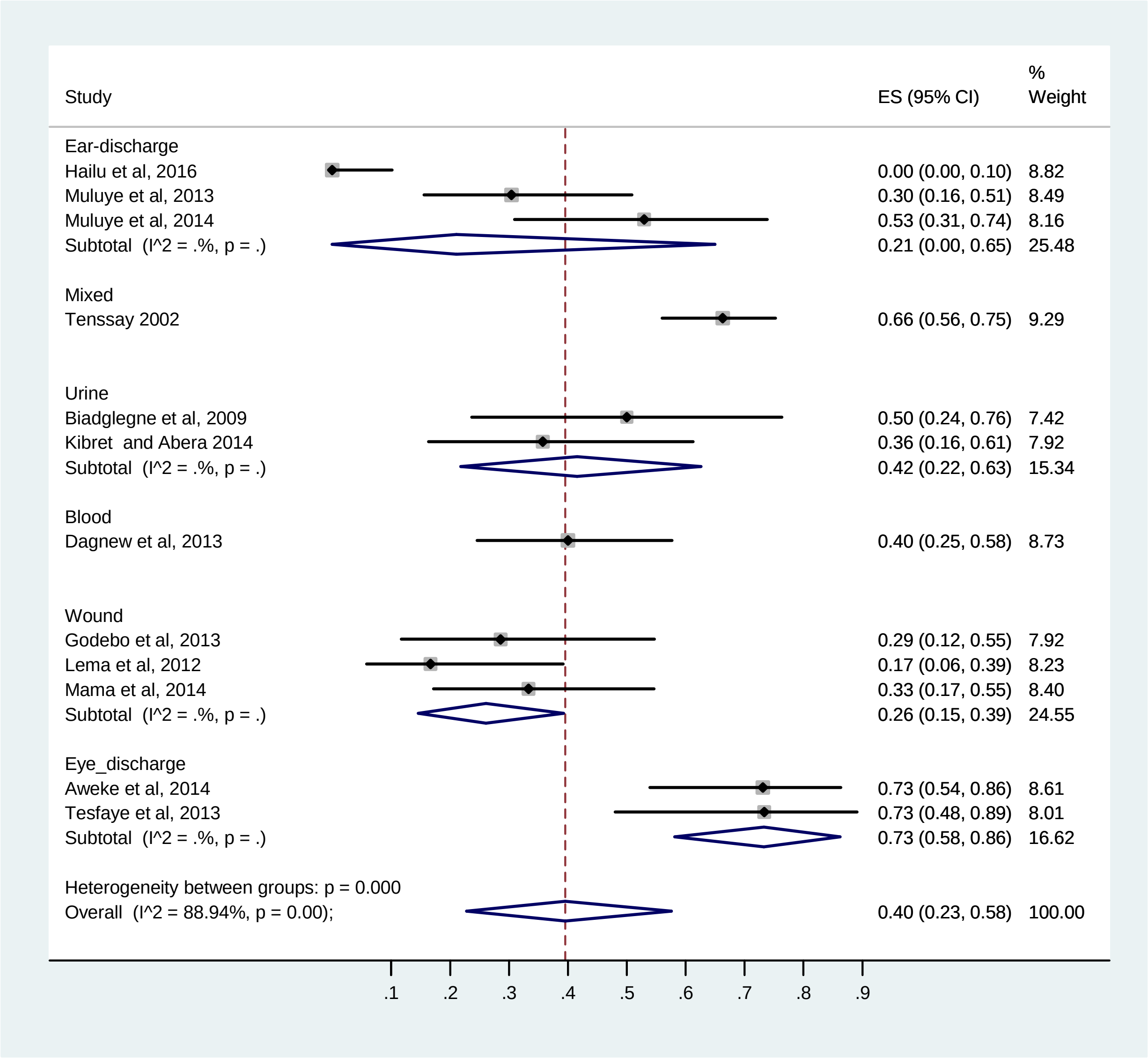
Study
ES (95% CI)
%
Weight
Ear-discharge
Hailu et al, 2016
0.00 (0.00, 0.10)
8.82
Muluye et al, 2013
0.30 (0.16, 0.51)
8.49
Muluye et al, 2014
0.53 (0.31, 0.74)
8.16
Subtotal (I^2 = .%, p = .)
0.21 (0.00, 0.65)
25.48
Mixed
Tenssay 2002
0.66 (0.56, 0.75)
9.29
Urine
Biadglegne et al, 2009
0.50 (0.24, 0.76)
7.42
Kibret and Abera 2014
0.36 (0.16, 0.61)
7.92
Subtotal (I^2 = .%, p = .)
0.42 (0.22, 0.63)
15.34
Blood
Dagnew et al, 2013
0.40 (0.25, 0.58)
8.73
Wound
Godebo et al, 2013
0.29 (0.12, 0.55)
7.92
Lema et al, 2012
0.17 (0.06, 0.39)
8.23
Mama et al, 2014
0.33 (0.17, 0.55)
8.40
Subtotal (I^2 = .%, p = .)
0.26 (0.15, 0.39)
24.55
Eye_discharge
Aweke et al, 2014
0.73 (0.54, 0.86)
8.61
Tesfaye et al, 2013
0.73 (0.48, 0.89)
8.01
Subtotal (I^2 = .%, p = .)
0.73 (0.58, 0.86)
16.62
Heterogeneity between groups: p = 0.000
Overall (I^2 = 88.94%, p = 0.00);
0.40 (0.23, 0.58)
100.00
.1
.2
.3
.4
.5
.6
.7
.8
.9
