# Supplementary material for: Prevalence and antimicrobial resistance of coagulase negative staphylococci clinical isolates from Ethiopia: a meta-analysis
Source: BMC Microbiol. 2018 May 25;18:43. doi: 10.1186/s12866-018-1188-6 (PMC5970528; doi:10.1186/s12866-018-1188-6)
Supplement: Supplementary file 5 [file 12866_2018_1188_MOESM14_ESM.docx]

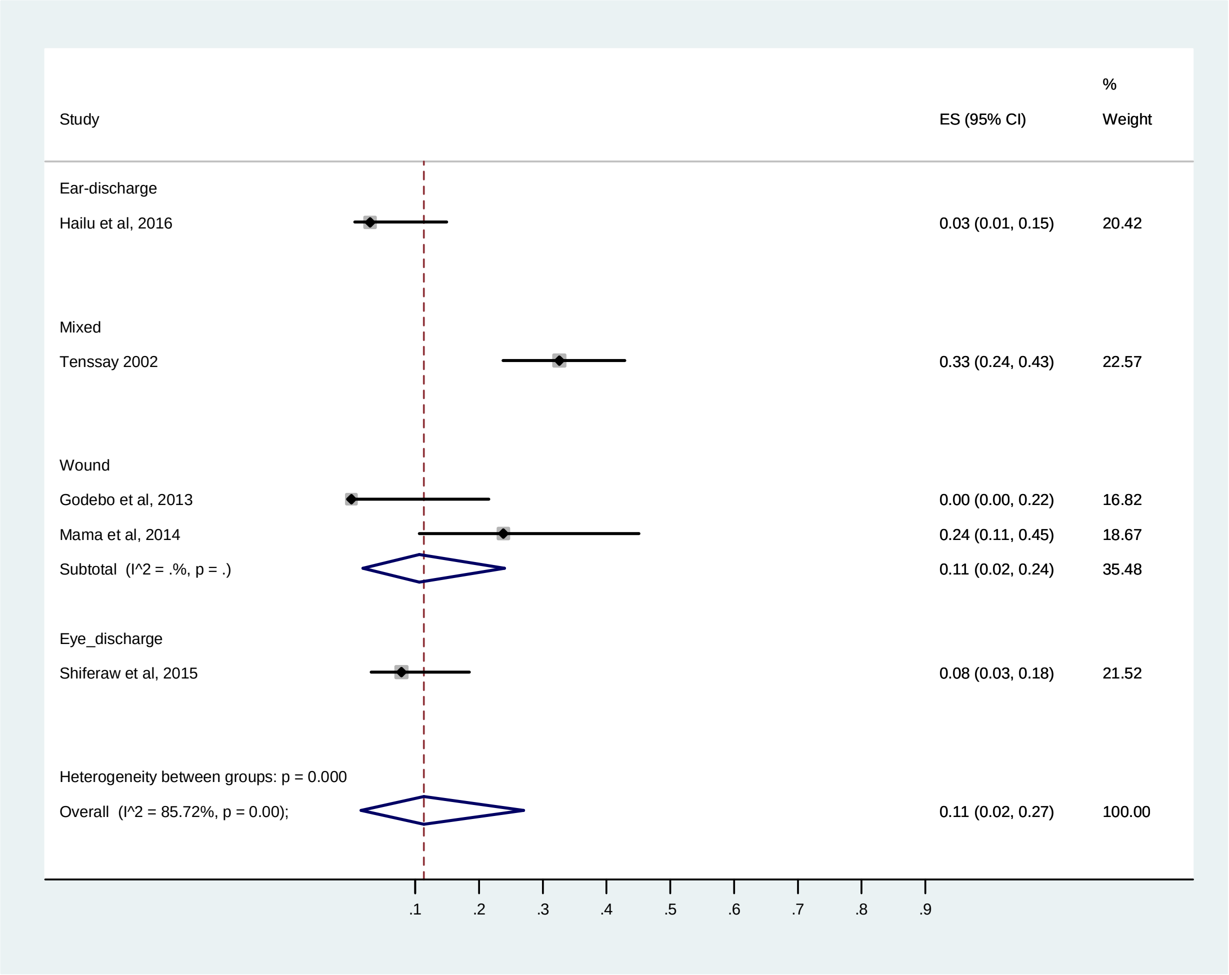

Study
ES (95% CI)
%
Weight
Ear-discharge
Hailu et al, 2016
0.03 (0.01, 0.15)
20.42
Mixed
Tenssay 2002
0.33 (0.24, 0.43)
22.57
Wound
Godebo et al, 2013
0.00 (0.00, 0.22)
16.82
Mama et al, 2014
0.24 (0.11, 0.45)
18.67
Subtotal (I^2 = .%, p = .)
0.11 (0.02, 0.24)
35.48
Eye_discharge
Shiferaw et al, 2015
0.08 (0.03, 0.18)
21.52
Heterogeneity between groups: p = 0.000
Overall (I^2 = 85.72%, p = 0.00);
0.11 (0.02, 0.27)
100.00
.1
.2
.3
.4
.5
.6
.7
.8
.9
