# Supplementary material for: Prevalence and antimicrobial resistance of coagulase negative staphylococci clinical isolates from Ethiopia: a meta-analysis
Source: BMC Microbiol. 2018 May 25;18:43. doi: 10.1186/s12866-018-1188-6 (PMC5970528; doi:10.1186/s12866-018-1188-6)
Supplement: Supplementary file 6 [file 12866_2018_1188_MOESM15_ESM.docx]

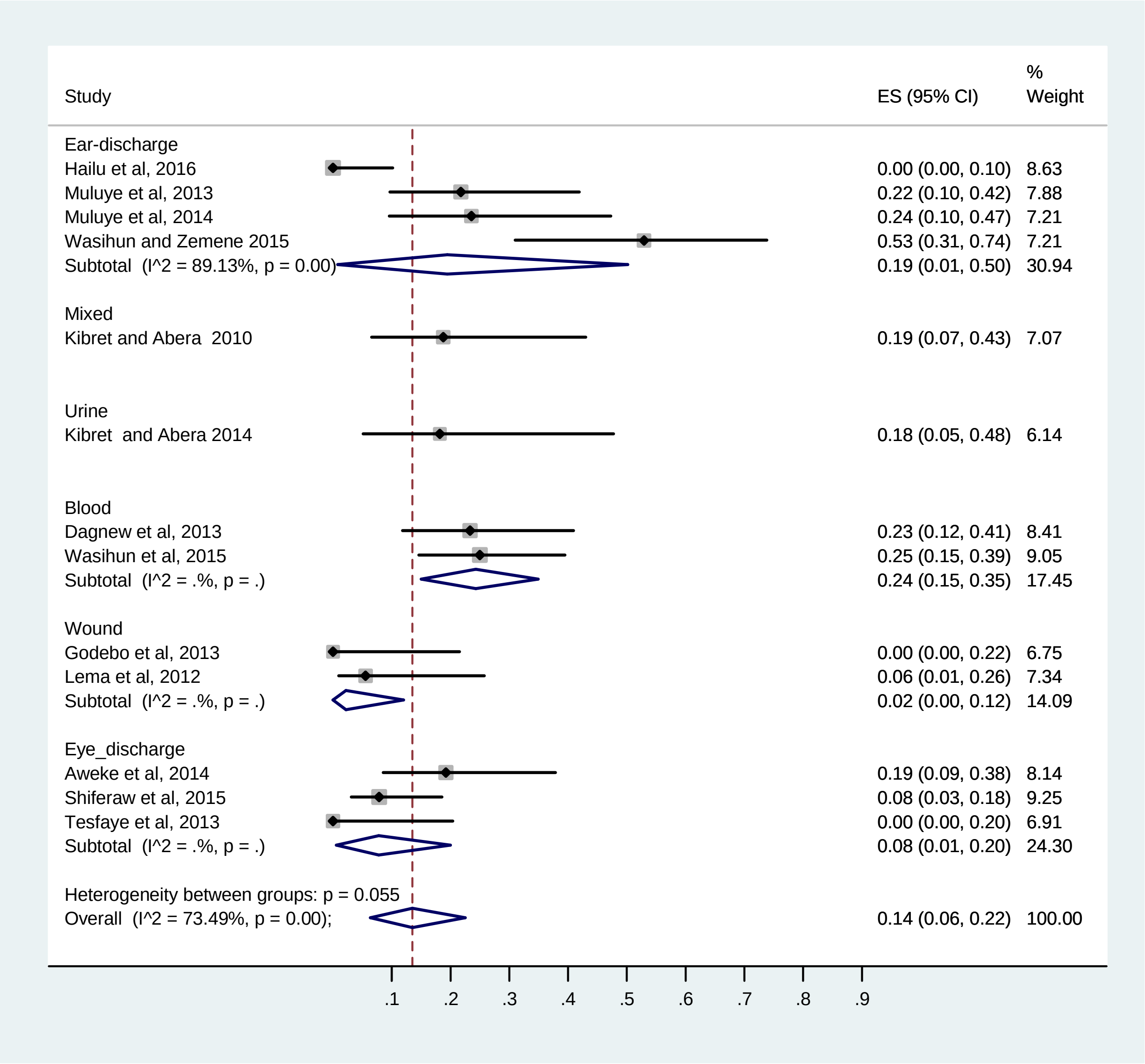
Study
ES (95% CI)
% Weight
Ear-discharge
Hailu et al, 2016
Muluye et al, 2013
Muluye et al, 2014
Wasihun and Zemene 2015
Subtotal (I^2 = 89.13%, p = 0.00)
Mixed
Kibret and Abera 2010
Urine
Kibret and Abera 2014
Blood
Dagnew et al, 2013
Wasihun et al, 2015
Subtotal (I^2 = .%, p = .)
Wound
Godebo et al, 2013
Lema et al, 2012
Subtotal (I^2 = .%, p = .)
Eye_discharge
Aweke et al, 2014
Shiferaw et al, 2015
Tesfaye et al, 2013
Subtotal (I^2 = .%, p = .)
Heterogeneity between groups: p = 0.055
Overall (I^2 = 73.49%, p = 0.00);
0.00 (0.00, 0.10) 8.63
0.22 (0.10, 0.42) 7.88
0.24 (0.10, 0.47) 7.21
0.53 (0.31, 0.74) 7.21
0.19 (0.01, 0.50) 30.94
0.19 (0.07, 0.43) 7.07
0.18 (0.05, 0.48) 6.14
0.23 (0.12, 0.41) 8.41
0.25 (0.15, 0.39) 9.05
0.24 (0.15, 0.35) 17.45
0.00 (0.00, 0.22) 6.75
0.06 (0.01, 0.26) 7.34
0.02 (0.00, 0.12) 14.09
0.19 (0.09, 0.38) 8.14
0.08 (0.03, 0.18) 9.25
0.00 (0.00, 0.20) 6.91
0.08 (0.01, 0.20) 24.30
0.14 (0.06, 0.22) 100.00
.1
.2
.3
.4
.5
.6
.7
.8
.9
