# Supplementary material for: Prevalence and antimicrobial resistance of coagulase negative staphylococci clinical isolates from Ethiopia: a meta-analysis
Source: BMC Microbiol. 2018 May 25;18:43. doi: 10.1186/s12866-018-1188-6 (PMC5970528; doi:10.1186/s12866-018-1188-6)
Supplement: Supplementary file 7 [file 12866_2018_1188_MOESM16_ESM.docx]

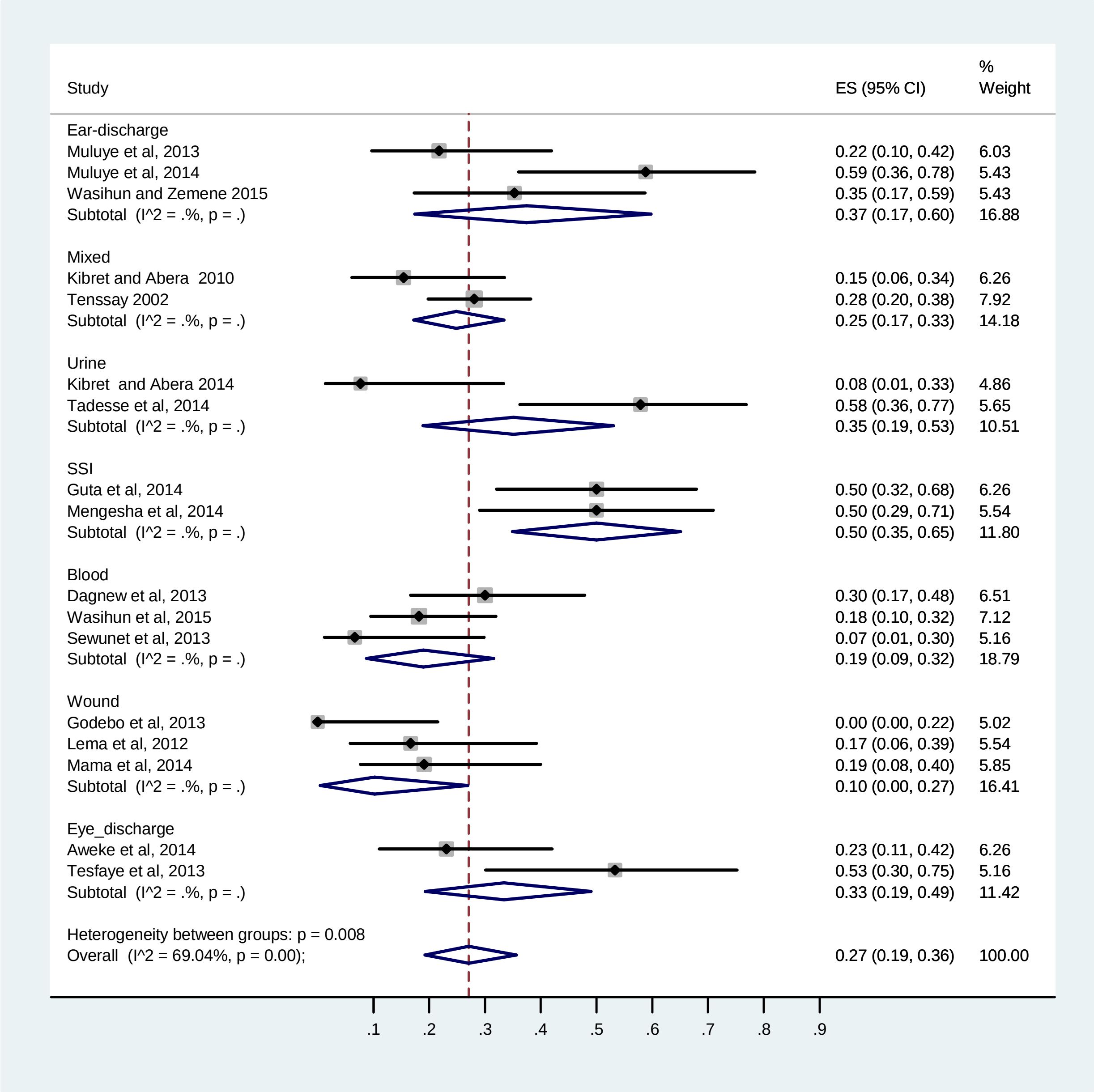
Study
ES (95% CI)
% Weight
Ear-discharge
Muluye et al, 2013 0.22 (0.10, 0.42) 6.03
Muluye et al, 2014 0.59 (0.36, 0.78) 5.43
Wasihun and Zemene 2015 0.35 (0.17, 0.59) 5.43
Subtotal (I^2 = .%, p = .) 0.37 (0.17, 0.60) 16.88
Mixed
Kibret and Abera 2010 0.15 (0.06, 0.34) 6.26
Tenssay 2002 0.28 (0.20, 0.38) 7.92
Subtotal (I^2 = .%, p = .) 0.25 (0.17, 0.33) 14.18
Urine
Kibret and Abera 2014 0.08 (0.01, 0.33) 4.86
Tadesse et al, 2014 0.58 (0.36, 0.77) 5.65
Subtotal (I^2 = .%, p = .) 0.35 (0.19, 0.53) 10.51
SSI
Guta et al, 2014 0.50 (0.32, 0.68) 6.26
Mengesha et al, 2014 0.50 (0.29, 0.71) 5.54
Subtotal (I^2 = .%, p = .) 0.50 (0.35, 0.65) 11.80
Blood
Dagnew et al, 2013 0.30 (0.17, 0.48) 6.51
Wasihun et al, 2015 0.18 (0.10, 0.32) 7.12
Sewunet et al, 2013 0.07 (0.01, 0.30) 5.16
Subtotal (I^2 = .%, p = .) 0.19 (0.09, 0.32) 18.79
Wound
Godebo et al, 2013 0.00 (0.00, 0.22) 5.02
Lema et al, 2012 0.17 (0.06, 0.39) 5.54
Mama et al, 2014 0.19 (0.08, 0.40) 5.85
Subtotal (I^2 = .%, p = .) 0.10 (0.00, 0.27) 16.41
Eye_discharge
Aweke et al, 2014 0.23 (0.11, 0.42) 6.26
Tesfaye et al, 2013 0.53 (0.30, 0.75) 5.16
Subtotal (I^2 = .%, p = .) 0.33 (0.19, 0.49) 11.42
Heterogeneity between groups: p = 0.008
Overall (I^2 = 69.04%, p = 0.00); 0.27 (0.19, 0.36) 100.00
.1
.2
.3
.4
.5
.6
.7
.8
.9
