# Supplementary material for: Prevalence and antimicrobial resistance of coagulase negative staphylococci clinical isolates from Ethiopia: a meta-analysis
Source: BMC Microbiol. 2018 May 25;18:43. doi: 10.1186/s12866-018-1188-6 (PMC5970528; doi:10.1186/s12866-018-1188-6)
Supplement: Supplementary file 8 [file 12866_2018_1188_MOESM17_ESM.docx]

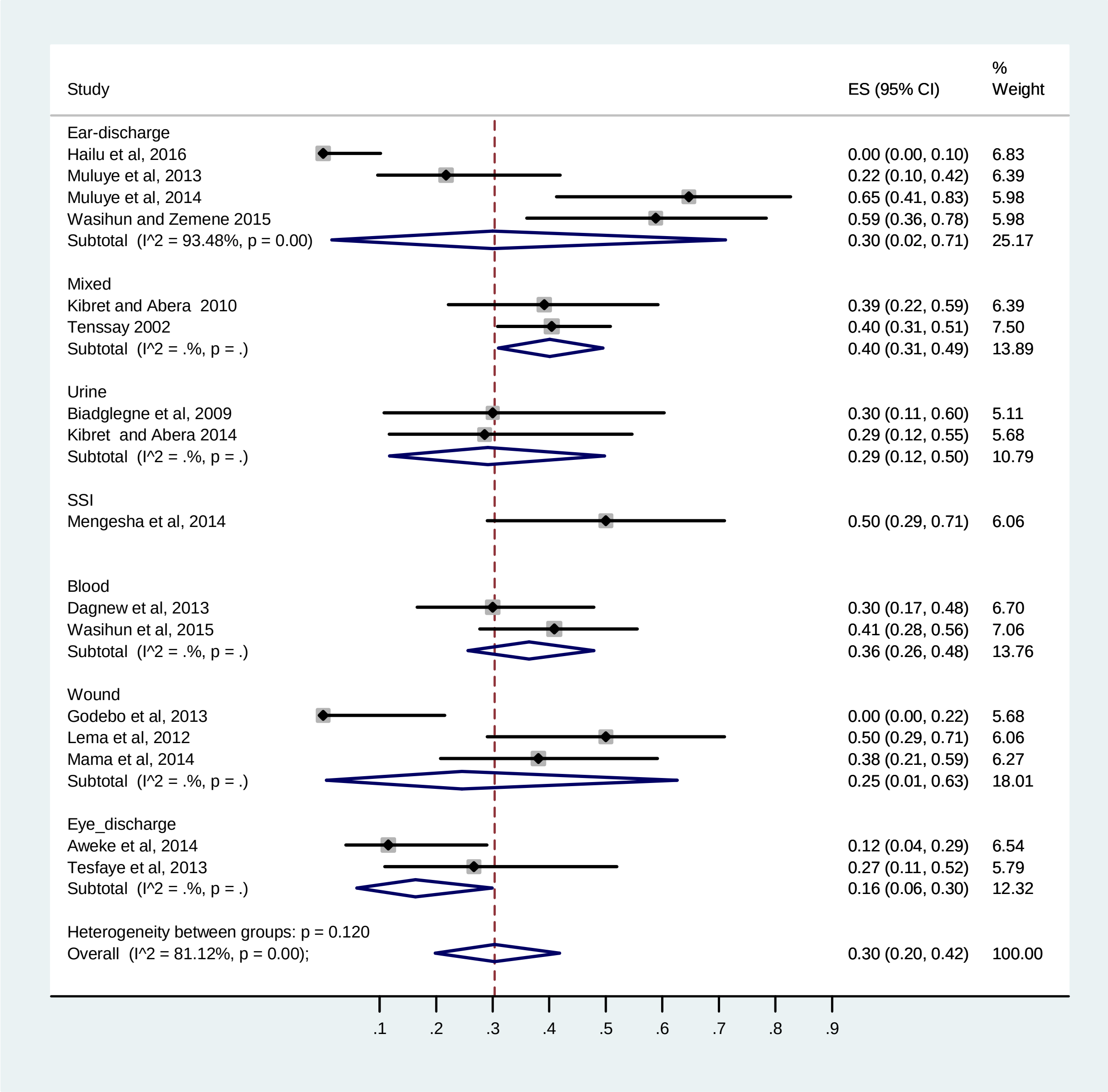

Study
ES (95% CI)
%
Weight
Ear-discharge
Hailu et al, 2016 0.00 (0.00, 0.10) 6.83
Muluye et al, 2013 0.22 (0.10, 0.42) 6.39
Muluye et al, 2014 0.65 (0.41, 0.83) 5.98
Wasihun and Zemene 2015 0.59 (0.36, 0.78) 5.98
Subtotal (I^2 = 93.48%, p = 0.00) 0.30 (0.02, 0.71) 25.17
Mixed
Kibret and Abera 2010 0.39 (0.22, 0.59) 6.39
Tenssay 2002 0.40 (0.31, 0.51) 7.50
Subtotal (I^2 = .%, p = .) 0.40 (0.31, 0.49) 13.89
Urine
Biadglegne et al, 2009 0.30 (0.11, 0.60) 5.11
Kibret and Abera 2014 0.29 (0.12, 0.55) 5.68
Subtotal (I^2 = .%, p = .) 0.29 (0.12, 0.50) 10.79
SSI
Mengesha et al, 2014 0.50 (0.29, 0.71) 6.06
Blood
Dagnew et al, 2013 0.30 (0.17, 0.48) 6.70
Wasihun et al, 2015 0.41 (0.28, 0.56) 7.06
Subtotal (I^2 = .%, p = .) 0.36 (0.26, 0.48) 13.76
Wound
Godebo et al, 2013 0.00 (0.00, 0.22) 5.68
Lema et al, 2012 0.50 (0.29, 0.71) 6.06
Mama et al, 2014 0.38 (0.21, 0.59) 6.27
Subtotal (I^2 = .%, p = .) 0.25 (0.01, 0.63) 18.01
Eye_discharge
Aweke et al, 2014 0.12 (0.04, 0.29) 6.54
Tesfaye et al, 2013 0.27 (0.11, 0.52) 5.79
Subtotal (I^2 = .%, p = .) 0.16 (0.06, 0.30) 12.32
Heterogeneity between groups: p = 0.120
Overall (I^2 = 81.12%, p = 0.00); 0.30 (0.20, 0.42) 100.00
.1 .2 .3 .4 .5 .6 .7 .8 .9
