# Supplementary material for: Prevalence and antimicrobial resistance of coagulase negative staphylococci clinical isolates from Ethiopia: a meta-analysis
Source: BMC Microbiol. 2018 May 25;18:43. doi: 10.1186/s12866-018-1188-6 (PMC5970528; doi:10.1186/s12866-018-1188-6)
Supplement: Supplementary file 9 [file 12866_2018_1188_MOESM1_ESM.docx]

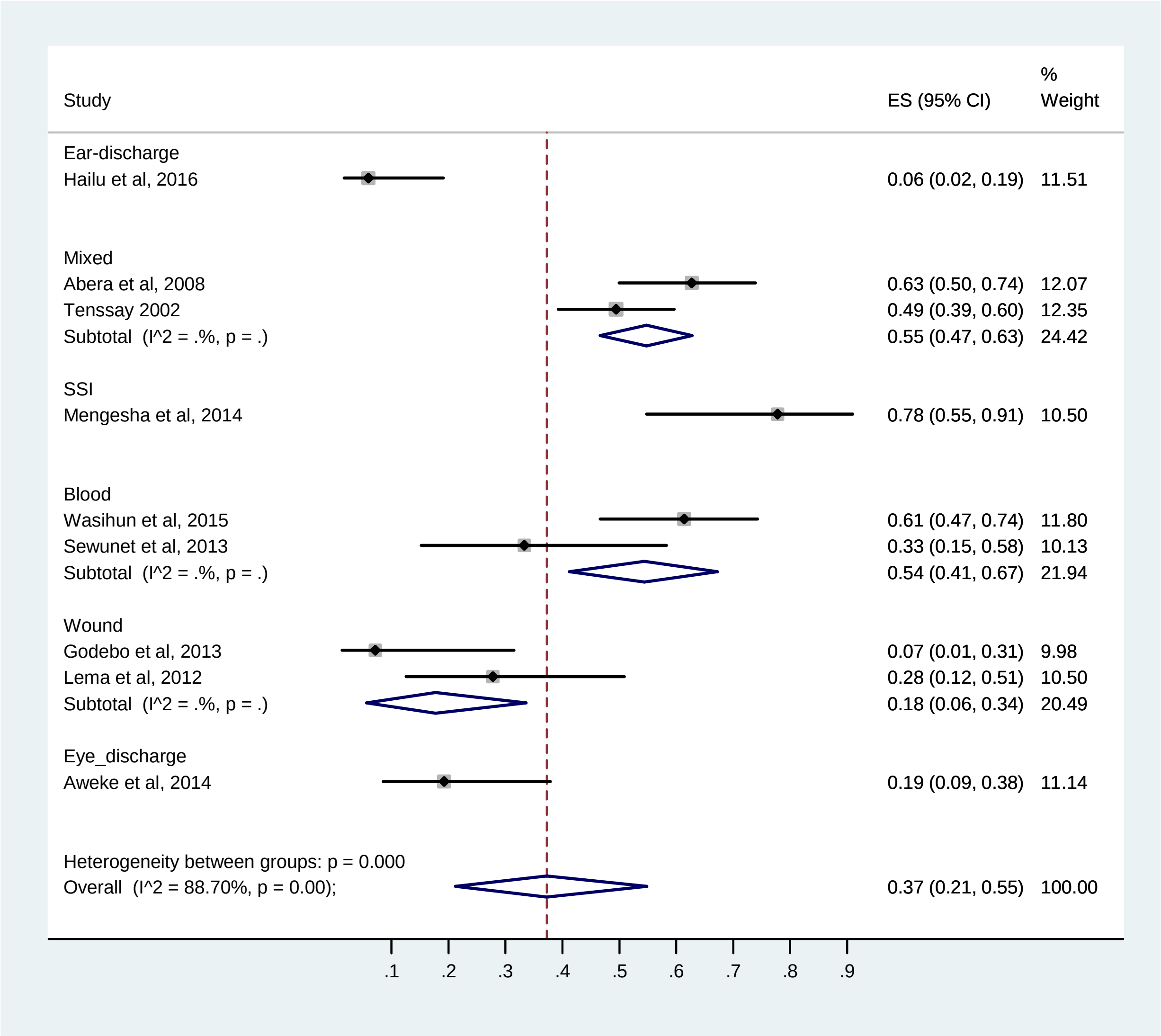

Study
ES (95% CI)
%
Weight
Ear-discharge
Hailu et al, 2016
0.06 (0.02, 0.19)
11.51
Mixed
Abera et al, 2008
0.63 (0.50, 0.74)
12.07
Tenssay 2002
0.49 (0.39, 0.60)
12.35
Subtotal (I^2 = .%, p = .)
0.55 (0.47, 0.63)
24.42
SSI
Mengesha et al, 2014
0.78 (0.55, 0.91)
10.50
Blood
Wasihun et al, 2015
0.61 (0.47, 0.74)
11.80
Sewunet et al, 2013
0.33 (0.15, 0.58)
10.13
Subtotal (I^2 = .%, p = .)
0.54 (0.41, 0.67)
21.94
Wound
Godebo et al, 2013
0.07 (0.01, 0.31)
9.98
Lema et al, 2012
0.28 (0.12, 0.51)
10.50
Subtotal (I^2 = .%, p = .)
0.18 (0.06, 0.34)
20.49
Eye_discharge
Aweke et al, 2014
0.19 (0.09, 0.38)
11.14
Heterogeneity between groups: p = 0.000
Overall (I^2 = 88.70%, p = 0.00);
0.37 (0.21, 0.55)
100.00
.1
.2
.3
.4
.5
.6
.7
.8
.9
