# Supplementary material for: Prevalence and antimicrobial resistance of coagulase negative staphylococci clinical isolates from Ethiopia: a meta-analysis
Source: BMC Microbiol. 2018 May 25;18:43. doi: 10.1186/s12866-018-1188-6 (PMC5970528; doi:10.1186/s12866-018-1188-6)
Supplement: Supplementary file 10 [file 12866_2018_1188_MOESM2_ESM.docx]

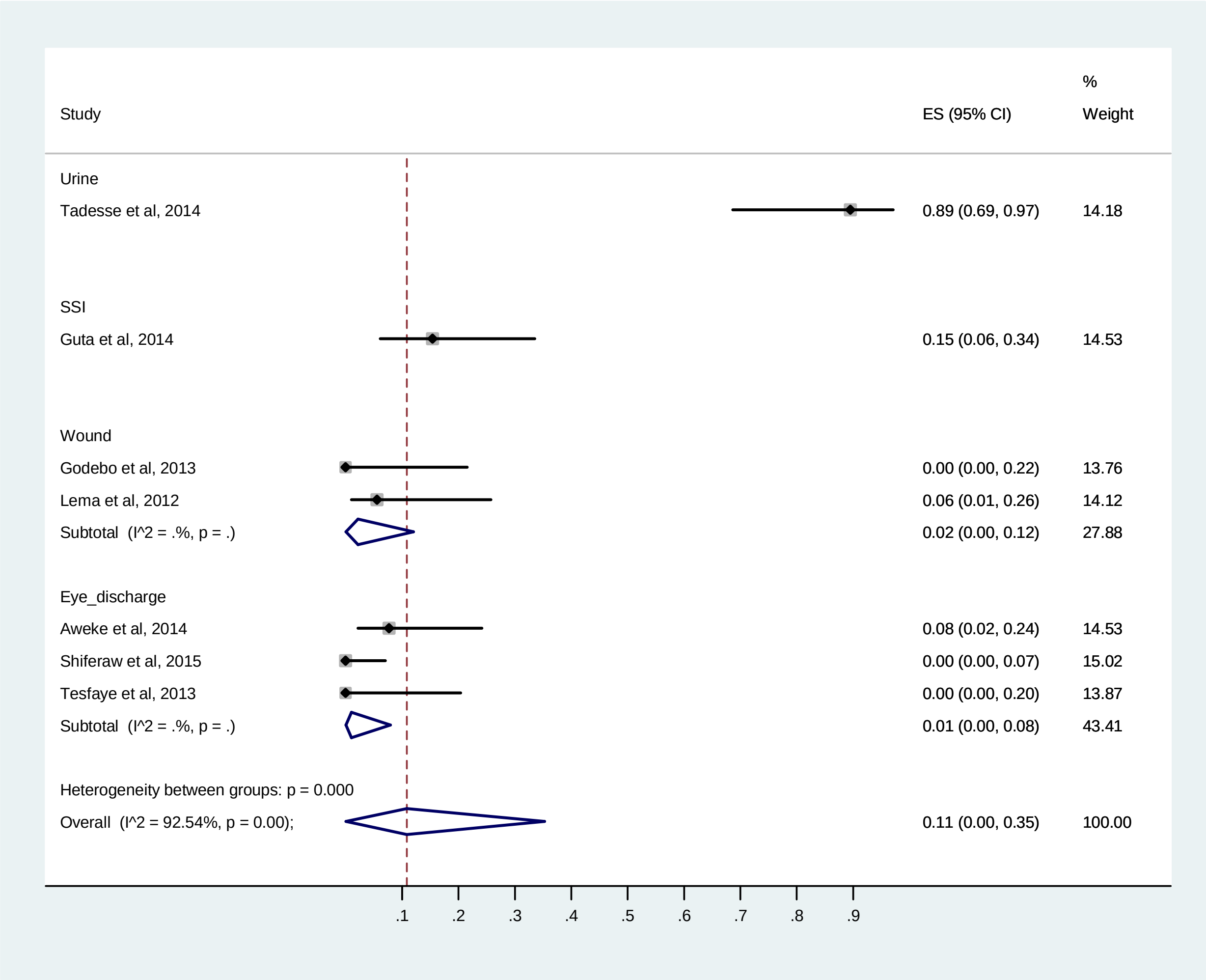

%
Study
ES (95% CI)
Weight
Urine
Tadesse et al, 2014
0.89 (0.69, 0.97)
14.18
SSI
Guta et al, 2014
0.15 (0.06, 0.34)
14.53
Wound
Godebo et al, 2013
0.00 (0.00, 0.22)
13.76
Lema et al, 2012
0.06 (0.01, 0.26)
14.12
Subtotal (I^2 = .%, p = .)
0.02 (0.00, 0.12)
27.88
Eye_discharge
Aweke et al, 2014
0.08 (0.02, 0.24)
14.53
Shiferaw et al, 2015
0.00 (0.00, 0.07)
15.02
Tesfaye et al, 2013
0.00 (0.00, 0.20)
13.87
Subtotal (I^2 = .%, p = .)
0.01 (0.00, 0.08)
43.41
Heterogeneity between groups: p = 0.000
Overall (I^2 = 92.54%, p = 0.00);
0.11 (0.00, 0.35)
100.00
.1
.2
.3
.4
.5
.6
.7
.8
.9
