# Supplementary material for: Prevalence and antimicrobial resistance of coagulase negative staphylococci clinical isolates from Ethiopia: a meta-analysis
Source: BMC Microbiol. 2018 May 25;18:43. doi: 10.1186/s12866-018-1188-6 (PMC5970528; doi:10.1186/s12866-018-1188-6)
Supplement: Supplementary file 12 [file 12866_2018_1188_MOESM4_ESM.docx]

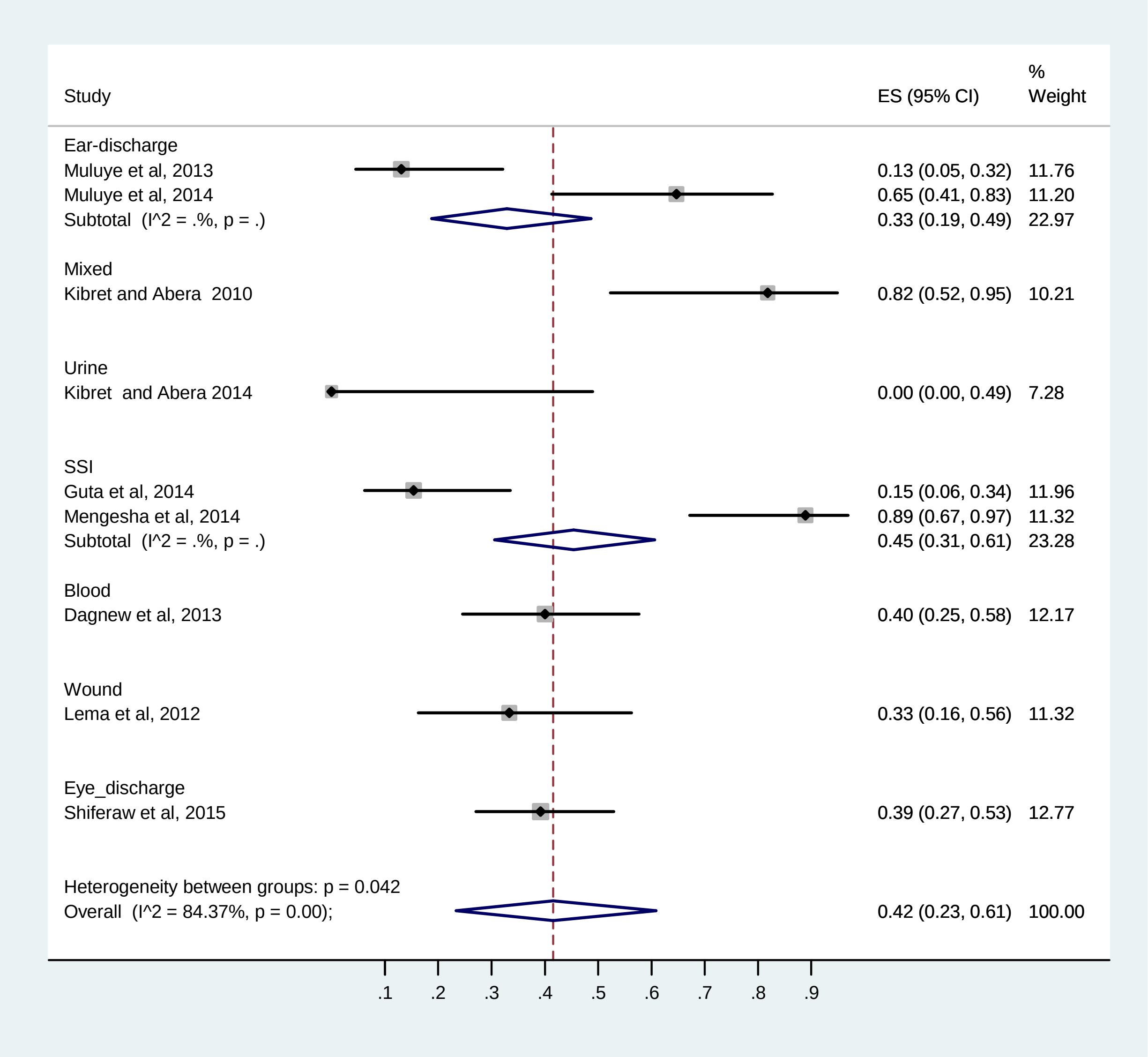

Study
ES (95% CI)
%
Weight
Ear-discharge
Muluye et al, 2013
0.13 (0.05, 0.32)
11.76
Muluye et al, 2014
0.65 (0.41, 0.83)
11.20
Subtotal (I^2 = .%, p = .)
0.33 (0.19, 0.49)
22.97
Mixed
Kibret and Abera 2010
0.82 (0.52, 0.95)
10.21
Urine
Kibret and Abera 2014
0.00 (0.00, 0.49)
7.28
SSI
Guta et al, 2014
0.15 (0.06, 0.34)
11.96
Mengesha et al, 2014
0.89 (0.67, 0.97)
11.32
Subtotal (I^2 = .%, p = .)
0.45 (0.31, 0.61)
23.28
Blood
Dagnew et al, 2013
0.40 (0.25, 0.58)
12.17
Wound
Lema et al, 2012
0.33 (0.16, 0.56)
11.32
Eye_discharge
Shiferaw et al, 2015
0.39 (0.27, 0.53)
12.77
Heterogeneity between groups: p = 0.042
Overall (I^2 = 84.37%, p = 0.00);
0.42 (0.23, 0.61)
100.00
.1
.2
.3
.4
.5
.6
.7
.8
.9
