# Supplementary material for: Prevalence and antimicrobial resistance of coagulase negative staphylococci clinical isolates from Ethiopia: a meta-analysis
Source: BMC Microbiol. 2018 May 25;18:43. doi: 10.1186/s12866-018-1188-6 (PMC5970528; doi:10.1186/s12866-018-1188-6)
Supplement: Supplementary file 13 [file 12866_2018_1188_MOESM5_ESM.docx]

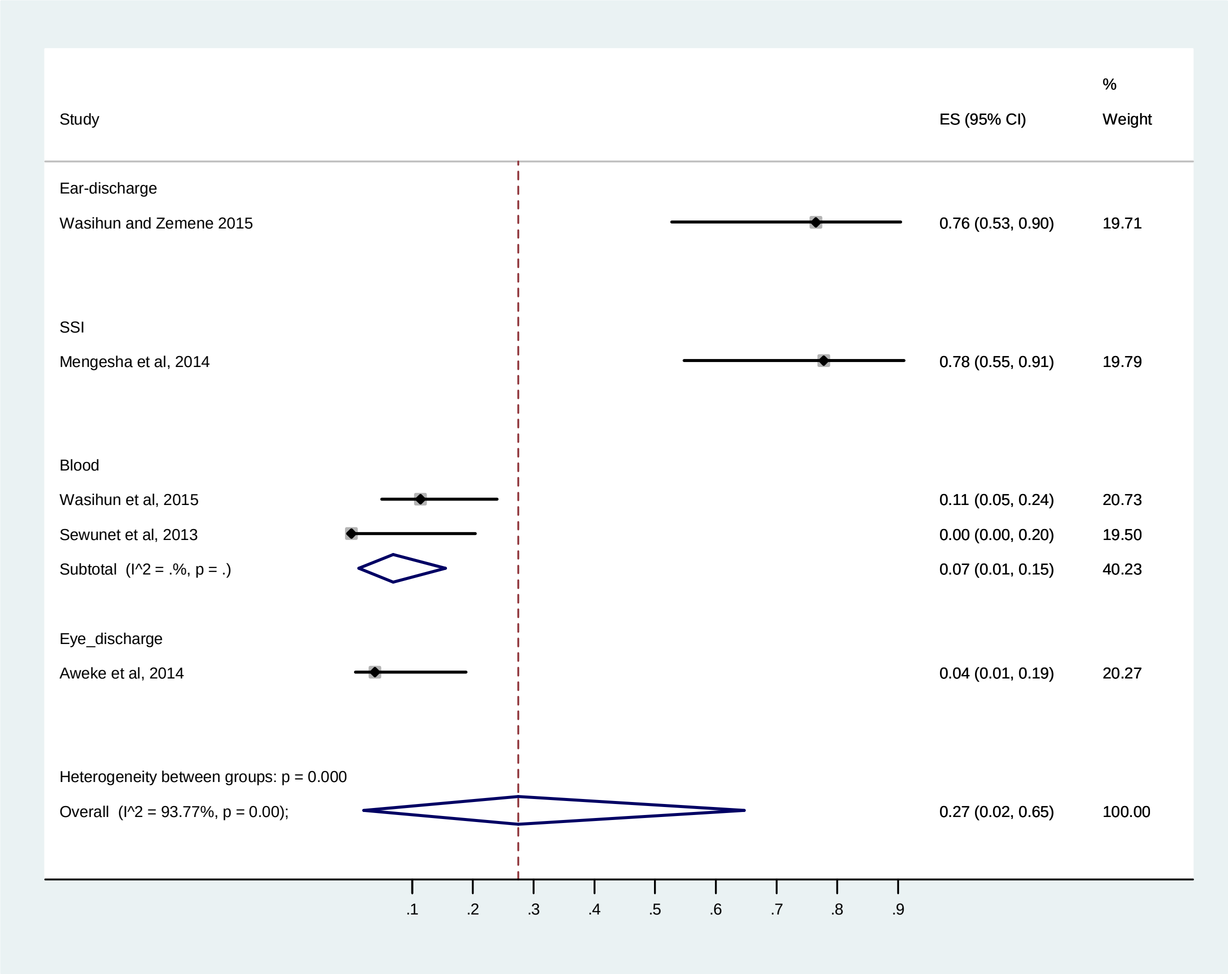

Study
ES (95% CI)
%
Weight
Ear-discharge
Wasihun and Zemene 2015
0.76 (0.53, 0.90)
19.71
SSI
Mengesha et al, 2014
0.78 (0.55, 0.91)
19.79
Blood
Wasihun et al, 2015
0.11 (0.05, 0.24)
20.73
Sewunet et al, 2013
0.00 (0.00, 0.20)
19.50
Subtotal (I^2 = .%, p = .)
0.07 (0.01, 0.15)
40.23
Eye_discharge
Aweke et al, 2014
0.04 (0.01, 0.19)
20.27
Heterogeneity between groups: p = 0.000
Overall (I^2 = 93.77%, p = 0.00);
0.27 (0.02, 0.65)
100.00
.1
.2
.3
.4
.5
.6
.7
.8
.9
