# Supplementary material for: Prevalence and antimicrobial resistance of coagulase negative staphylococci clinical isolates from Ethiopia: a meta-analysis
Source: BMC Microbiol. 2018 May 25;18:43. doi: 10.1186/s12866-018-1188-6 (PMC5970528; doi:10.1186/s12866-018-1188-6)
Supplement: Supplementary file 16 [file 12866_2018_1188_MOESM8_ESM.docx]

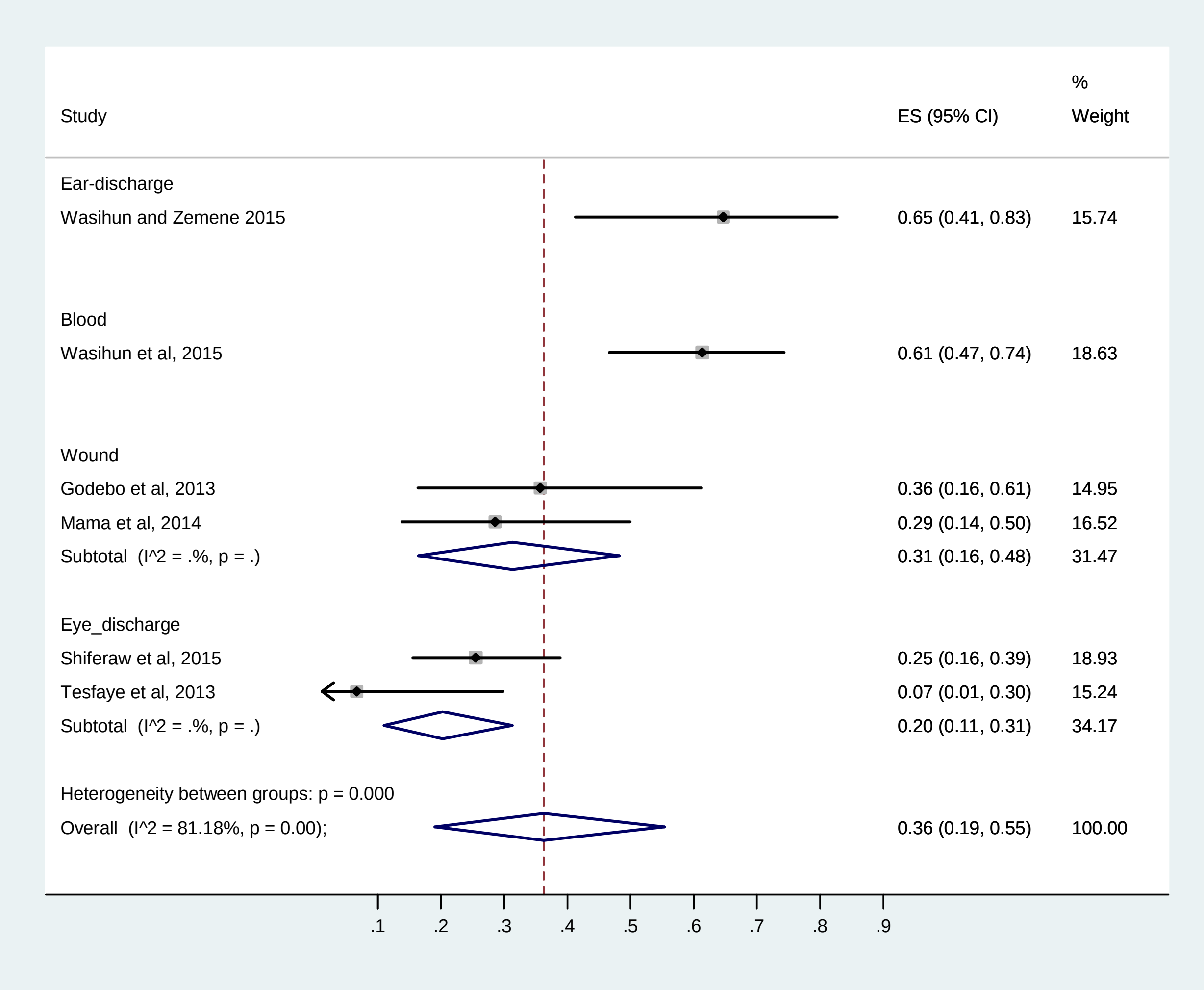
Study
ES (95% CI)
%
Weight
Ear-discharge
Wasihun and Zemene 2015
0.65 (0.41, 0.83)
15.74
Blood
Wasihun et al, 2015
0.61 (0.47, 0.74)
18.63
Wound
Godebo et al, 2013
0.36 (0.16, 0.61)
14.95
Mama et al, 2014
0.29 (0.14, 0.50)
16.52
Subtotal (I^2 = .%, p = .)
0.31 (0.16, 0.48)
31.47
Eye_discharge
Shiferaw et al, 2015
0.25 (0.16, 0.39)
18.93
Tesfaye et al, 2013
0.07 (0.01, 0.30)
15.24
Subtotal (I^2 = .%, p = .)
0.20 (0.11, 0.31)
34.17
Heterogeneity between groups: p = 0.000
Overall (I^2 = 81.18%, p = 0.00);
0.36 (0.19, 0.55)
100.00
.1
.2
.3
.4
.5
.6
.7
.8
.9
